# Antireflective nanocoatings for UV-sensation: the case of predatory owlfly insects

**DOI:** 10.1186/s12951-017-0287-0

**Published:** 2017-07-14

**Authors:** Mikhail Kryuchkov, Jannis Lehmann, Jakob Schaab, Manfred Fiebig, Vladimir L. Katanaev

**Affiliations:** 1 Department of Pharmacology and Toxicology, University of Lausanne, Rue du Bugnon 27, 1011 Lausanne, Switzerland; 2 Department of Materials, ETH Zurich, Vladimir-Prelog-Weg 4, 8093 Zurich, Switzerland; 3 School of Biomedicine, Far Eastern Federal University, Sukhanova Street 8, Vladivostok, 690922 Russian Federation

## Abstract

Moth-eye nanostructures, discovered to coat corneae of certain nocturnal insects, have inspired numerous technological applications to reduce light reflectance from solar cells, light-emitting diodes, and optical detectors. Technological developments require such nanocoatings to possess broadband antireflective properties, transcending the visual light spectrum, in which animals typically operate. Here we describe the corneal nanostructures of the visual organ exclusive in UV sensation of the hunting insect *Libelloides macaronius* and report their supreme anti-light-reflectance capacity.

The owlfly (Insecta: Neuroptera) *Libelloides macaronius* (Scopoli 1763) leads a daytime predatory lifestyle and possesses hunting-specialized eyes. These eyes are divided into 2 parts: the upper dorsofrontal (DF) and the lower ventrolateral (VL) (Fig. 1a, b). Curiously, the DF part is of the optical superposition type characteristic of nocturnal insects, and further contains only one rhodopsin with absorption peak at 345 nm. These adaptations optimize the task of hunting for small insects contrasting against the sky, as hunting in the UV range limits diffraction, increasing the resolving power of the eye and thus the distance at which the prey can be found. At the same time, given the low intensity of the UV light reaching the ground (only 3–4% of the total light spectrum), these adaptations serve to maximize the UV light reception. In contrast to the DF eye, the VL part has all the specifics of a normal daytime insect’s eye [1, 2]. Nocturnal insects such as moths have an additional way of increasing the light transmission through their corneal lenses, using the phenomenon called the “moth eye” effect [3, 4]. This effect is based on the principle that a surface coated with particular nanostructures may reflect less light than a smooth surface [5].

**Fig. 1:**
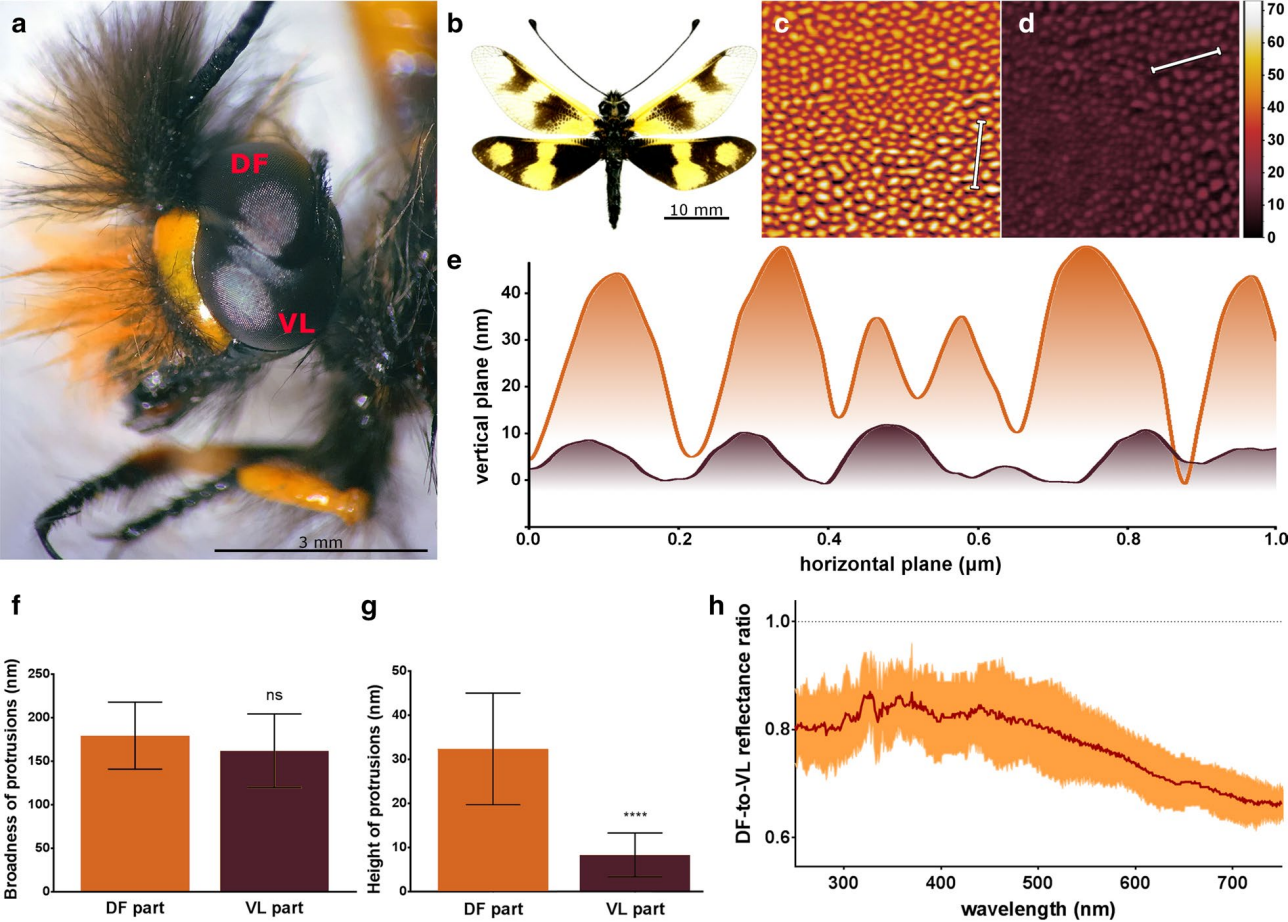
Dorsofrontal and ventrolateral parts of *L. macaronius* eyes possess different corneal nanocoatings, resulting in different light reflectance properties. **a** Light microscopy (lateral view) of a *L. macaronius* head. The furrow dividing the ventrolateral (VL) from the dorsofrontal (DF) parts can be seen. **b** Photograph of an owlfly (photo kindly provided by T. B. Bersatu from http://www.thebugmaniac.com). **c** and **d** Representative AFM scans of corneal surfaces of the DF (**c**) and VL (**d**) eyes reveal clear difference between the nanostructural coatings of the two eye parts. The surface height (in nm) is indicated by the color scale shown next to the images; the minimum level is set to zero for both scans. Both squares are 4 × 4 μm and in the same height color scale. **e** Cross-sectional profiles of DF (in *orange*) and VL (in *brown*) cornea of 1 µm length. The location of the cross-sectioned zones is indicated by the *white bars* on the AFM scans above. **f** and **g** Measurements of the broadness (**f**) and the height (**g**) of protrusions from the DF (*orange*) and VL (*brown*) eye parts. Data are shown as mean ± SD, n = 20. Student’s t test was used to assess statistical significance, “ns” stands for non-significant, “****” indicates the p value ≤ 0.0001. **h** Ratio of the reflection spectra (from 250 to 750 nm) measured for the DF and VL eye parts. Data are shown as mean (in *red*) ± SD (in *orange*), n = 2. The *spotted line* shows the ratio of 1.0 (no reflection difference) across the spectrum

There are two basic ways to achieve this. One is that the incident light is trapped by multiple reflections on the structured surface, effectively guiding the light to the photo-sensitive part. Alternatively, a gradient of the optical refractive index created by the nanostructured surface bends the incident light towards the cornea. Technologically, these principles have inspired nanostructured arrays with broadband, quasi-omni-directional antireflective properties that are put to use in solar cells, light-emitting diodes and optical detectors [5].

Our previous studies have shown that nocturnal neuropteran *Chrysoperla carnea* has “merging nipples” nanostructures of about 20 nm in height [4]. Regarding owlflies with their daytime lifestyle, one could expect several possible types of the corneal nanocoatings. On one hand, the complete absence of nanostructures or the presence of degenerative dragonfly-like or wasp-like structures typical of daytime predators [4] could be expected. On the other hand, given the need to maximize the UV reception, one might also expect that the UV sensitive parts of the owlflies’ eyes should be coated with functional antireflective nanostructures. In this study, we discover that the UV-responsive DF part of the eyes of these insects indeed harbors elaborated nanocoatings, unlike the VL part. Correlating with this, we find that the DF part is significantly less reflecting than the VL part.

We used atomic force microscopy (AFM) for investigation of nanostructures coating the VL and DF parts of the owlflies’ eyes. Our analysis reveals that both parts have structures, which we previously called “merging nipples” [4]. However, despite the similar broadness of the nanostructures (Fig. 1e, f), the upper (DF) eyes show prominent protrusions with the height from 15 to 50 nm (32 nm in mean) (Fig. 1c, e, g), while the height of nanostructures from the VL eyes is maximally 18 nm (mean being 8 nm, Fig. 1d, e, g).

The height and aspect ratio of the protrusions, as well as the distance between them influence the antireflective properties of the nanostructures [5]. As the broadness and the inter-nipple distances of protrusions of the DF and VL parts were the same, the superior height of the DF nanostructures can be expected to augment the corneal transparency for the incident light of the wavelengths >250 nm (the maximal broadness of the owlflies’ nanostructures, Fig. 1c–g).

In order to directly test this expectation, we measured light reflection from the surface of the DF and VL eyes, finding that reflection from DF corneae is significantly lower than that from the VL corneae of owlflies (Fig. 1h). Curiously, this decrease in reflectance is seen not only in the UV but also in the visible part of the light spectrum (Fig. 1h).

Despite the long-accepted view that insect corneal nanocoatings serve the antireflective role [6, 7], direct experimental evidence in favor of this idea has in most cases been lacking. Together with our previous observation that nanocoatings-harboring overwater eyes of the whirligig beetles reflect less light than the smooth underwater eyes [8], this current communication on the optical properties of the owlflies’ eyes represents the second only direct proof of the antireflective function of insect nanocoatings.

## Methods

The dried samples of *L. macaronius* (mature adults from the Mersin region, Turkey) were obtained from the online shop http://www.thebugmaniac.com and guillotined, followed by removal of eyes from the head capsule with a scalpel and preparation for AFM as described previously [9, 10]. In brief, the cornea was separated from the retina by a combination of the gentle washing with water by a pipette and the physical detachment with a fine brush, followed by additional intensive washing in water. The resultant corneal samples were attached to a coverslip by a double-sided bonding tape. Microscopy images were collected in the contact mode with a scanning rate of 2.373 µm/sec on the NTegra-Prima AFM (NT-MDT), using long NSG11 cantilevers (NT-MDT) with the following specifications: resonance frequency 115–190 kHz, tip radius 10 nm, force constant 2.5–10 N/m. By applying the thermal tune method, a force constant of 9.6 ± 0.5 N/m was determined for the cantilever used for the scans presented in Fig. 1c, d. The Gwyddion software (Department of Nanometrology, Czech Metrology Institute) was used for visualization and quantification. The same samples were also used for reflectance measurements, using the JASCO MSV-370 micro-spectrophotometer in the reflection geometry. Using a non-dispersive Schwarzschild-objective and an aperture, the region of interest was set to an area of 300 × 300 µm spanning around 150 eye-facets. The spectral region from UV (250 nm) to near infrared (750 nm) was chosen to overspan the vision range of *L. macaronius.* The data is used to visualize the spectral ratio (R_(DF)_/R_(VL)_) between the two parts of the eyes (Fig. 1h).
